# Broadening the scope of multimodal precision prevention in Alzheimer’s disease research

**DOI:** 10.1007/s44192-026-00569-3

**Published:** 2026-08-11

**Authors:** Timothy Daly

**Affiliations:** 1 Bioethics Program, FLACSO Argentina, Tucumán 1966, C1050 AAN, Buenos Aires, Argentina; 2 CNRS UMR 8011, Science Norms Democracy, Sorbonne Université, Paris, France

**Keywords:** Alzheimer’s disease, Multimodal, Precision medicine, Translational science, Clinical translation

## Abstract

**Introduction:**

Within the last decade, translational research into Alzheimer’s disease and related dementias (ADRD) has embraced the complexity of disease mechanisms and pluralistic approaches to therapeutics. As part of this broader shift, the “multimodal precision prevention paradigm” has emerged as an actionable approach to reduce the public health burden of ADRD.

**Objective:**

To provide an overview of the definitions, scope, and limitations of the multimodal precision prevention paradigm for ADRD and propose a broader definition of translational research in ADRD research and policy.

**Methods:**

This article performs a critical analysis of leading proposals by different international research initiatives working on multimodal precision medicine for ADRD prevention.

**Main results:**

Within the multimodal precision prevention paradigm, two overlapping approaches are distinguished on a continuum: on one hand, researchers predominantly from the global North propose a multimodal therapy approach. On the other hand, authors from the global South are proposing a “multimodal diversity” approach stressing the need for a deeper understanding of, and action against, broader contributors to risk of ADRD. It is argued that the complexity of multimodal diversity requires extending the scope of what is typically called “translational research” beyond the “bench-to-bedside” metaphor to include broader policy entry points for prevention (such as action against the social determinants of ADRD).

**Conclusions:**

Translational research into ADRD faces a choice between narrow and broader policy entry points. This article suggests a broader definition of translational research as follows: translational research in Alzheimer’s disease and Alzheimer’s disease-related dementias should promote a multi-directional interaction between, and multimodal integration of, laboratory-based and diverse population research to achieve a robust, global understanding of brain health and disease that can inspire equitable public health policy and advance precision medicine.

## Introduction

According to the World Health Organization, Alzheimer’s disease (AD) is responsible for between 60 and 70% of cases of dementia, a progressive syndrome of cognitive decline and neuropsychiatric disturbances with no cure [1]. AD is associated with, and defined by, the accumulation of two signature proteins, amyloid-beta and tau proteins. The focus of this article is on what is known as AD and AD-related dementias (ADRD):

Alzheimer’s Disease and Alzheimer’s Disease Related Dementias (ADRD) refers to the most common forms of dementia. … While AD is the most common dementia diagnosis, ADRDs share many cognitive and pathological features with, and can be difficult to distinguish from, AD … more often than not, patients with a clinical diagnosis of Alzheimer’s disease have different mixtures of brain pathologies [2].

In the absence of effective treatment, translational research is understood as necessary to achieve the goal of reducing the public health burden of ADRD [3]. In 2015, Lon Schneider [3] defined translational research into ADRD as follows:

*research that plans to involve or includes humans and therapeutic interventions that range from preclinical to late stage trials and health technology assessment. The interventions may include drugs*,* biologicals*,* devices*,* and the full range of psychotherapeutic*,* psychosocial*,* and other non-pharmacological approaches*,* as well as technology assessment and effectiveness*,* services research*,* and clinical application … for Alzheimer disease*,* age-related cognitive impairment*,* and other conditions associated with cognitive impairment in late-life* (p. 91, [3]).

Over the past decade since 2015, the field of ADRD translational research has seen different trends in how AD is defined and diagnosed [4, 5], treated [6] and potentially prevented [7, 8]. This article focuses on a trend in translational research based on two simple ideas: using more than one treatment modality (e.g. a drug and a lifestyle intervention) at once, and tailoring interventions to the health needs of specific individuals and groups. It identifies two overlapping but divergent approaches within this paradigm, and discusses what their existence means for the future of translational ADRD research.

## The multimodal precision prevention paradigm for ADRD

This section will demonstrate that there is more than one approach within the broader paradigm of multimodal precision prevention. Proponents of both of the approaches described here, the “lifestyle/drug combination therapies” approach [9] that here are called multimodal therapy, and the “multimodal diversity” [10] approach, converge on the notions that multiple, tailored therapeutic approaches should be explored to address the public health burden of ADRD [9, 10]. However, as will be demonstrated, the multimodal therapy approach is much simpler and more limited in scope from the population perspective of reducing the public health burden of ADRD. Conversely, the complexity of the multimodal diversity scientific approach suffers from the difficulty of its implementation in a research and policy ecosystem that favours simplicity.

Before presenting both approaches, some definitions are useful to avoid confusion, given the related meaning of certain words: multidomain intervention; multimodal therapy; and precision prevention. A multidomain intervention means targeting several risk factors and disease mechanisms simultaneously, and generally refers to lifestyle interventions targeting diet, stimulation (physical, mental, social), and cardiovascular risk factors. Multimodal therapy means combining more than one therapeutic modality within the same intervention: for example, combining a drug with a (single or multidomain) lifestyle intervention. Precision prevention means anticipating and responding to the health needs of individuals based on their unique characteristics.

### The multimodal therapy approach

The last few decades of clinical research have seen the targeting of amyloid and tau in the brain of people with AD as well as other physiological processes [11]. Given the difficulties associated with this therapeutic strategy, there has been a general move towards a variety of drug targets within the AD drug development pipeline [12]. Moreover, there has been a surge of interest in the impact of lifestyle behaviours on cognitive health. Three major findings within these approaches have motivated multimodal therapeutics that combine action against modifiable risk factors and disease mechanisms simultaneously. First, the finding that up to 45% of cases of dementia are associated with modifiable risk factors such as low education, poor physical, mental and social health, and air pollution [8]. Second, successful randomised controlled trials of “multidomain interventions.” These so-called multidomain interventions target “vascular and lifestyle-related risk factors” [13] through improved nutrition, mental, physical and social activity, and cardiovascular risk monitoring. Such multidomain interventions also include the possibility of non-invasive brain stimulation [14]. Successful examples include the Finnish Geriatric Intervention Study to Prevent Cognitive Impairment and Disability (FINGER) and the U.S. study to protect brain health through lifestyle intervention to reduce risk (US POINTER) [15, 16]. Third, the approval of anti-amyloid medications in different health systems that suggest a modest impact on the slowing of cognitive decline in AD [6]. These suggest intuitively that by combining admittedly imperfect and limited therapeutics, more impact could be achieved.

In Europe, this therapeutic approach is being implemented in memory clinics through their re-orientation from diagnosis to multimodal prevention in an initiative called “Brain Health Services” [14]. The Worldwide FINGERS (WW-FINGERS) initiative is.

In summary, the multimodal therapy approach is fundamentally practical and aims to capitalise on existing data to maximise prevention of ADRD. Therefore, it does not primarily seek to deepen a theoretical understanding of brain health and dementia risk, but rather to apply what is already known or hypothesised about modifiable risk factors and brain stimulation to prevention. It is thus based on epidemiological correlations and small effect sizes from clinical trials, but it nevertheless has a research output, e.g. the results of clinical trials [9].

### The multimodal diversity approach

Even more recently, “multimodal diversity” [10], a “heuristic meta-model” to conceptualise global brain health, has emerged. This model embraces complexity. The specific concept of “multimodal diversity” is understood along three dimensions: data representation, multimodal integration, and computational metamodels.

Data representation stresses the need for diverse study populations, who have historically been underrepresented in clinical trials (e.g. ethnically diverse cohorts from lower-income countries). Multimodal integration combines neurological and psychological data with whole-body health indicators and environmental contextual factors to provide an overall indicator of brain health. Environmental factors, broadly construed, are collectively known as the “exposome,” the environmental exposures that an individual encounters throughout the life course. Groups within the multimodal diversity approach, particularly those in Latin America, are intensely studying the relationship between the exposome and cognitive decline [17, 18]. This has been made possible through computational metamodels, the application of machine learning to neuroimaging and other heterogeneous data, and has provided an avenue for detecting subtle age-related changes in brain structure and function to reconceptualise brain health [10]. This deepened understanding of brain health has revealed the influence of social determinants of health such as access to healthcare, pollution and different forms of privation [19] on brain health [17–19]. This expands the range of policy outputs available for ADRD prevention beyond a narrow focus on individual lifestyle choices that the multimodal therapy approach suffers from [20, 21].

Given its insistence on representativeness and the need for action against social determinants of health, the multimodal diversity approach is more likely to achieve brain health equity than the multimodal therapy approach.

### Comparing the two approaches

Table 1 provides a side-by-side, analytical comparison of the two approaches to multimodal precision prevention. Overall, the multimodal therapy approach is fundamentally a practice-first, knowledge-second approach. The multimodal diversity approach is a knowledge-first, practice-second approach that stresses the need for a solid theoretical grounding to guide fair public health action and precision medicine.

**Table 1:** Two different approaches within the multimodal precision prevention paradigm for Alzheimer’s disease and Alzheimer’s disease-related dementias (ADRD): multimodal therapy, a therapeutic approach, and the multimodal diversity approach, a scientific program with therapeutic consequences

Feature		
Covering term	Multimodal therapy	Multimodal diversity
Work within scope (not exclusive)	FINGER [15], FINGER 2.0 [9], Brain Health Services [14], Dementia prevention in memory clinics [22]	Exposome [17, 18], Ecological systems theory [23], Syndemics framework [24]
Primary aim	“prevention and risk reduction of cognitive impairment and dementia” [9]	“a comprehensive understanding of brain health” [10]
Secondary aim	Knowledge-generation through clinical trials [9, 15, 16]	“informing global health policies and advancing precision medicine” [10]
Methods	“multimodal therapy approaches targeting several modifiable risk factors and disease mechanisms simultaneously” [9]	“integrates biological, systemic, and environmental measures to generate more accurate, and individualized predictions” [10]
Evidence base	Lifestyle interpretation of modifiable risk [25] Falling prevalence of AD and dementia in high-income countries [26] Clinical trials with lifestyle and pharmacological interventions [9]	Ageing clocks in diverse populations [17, 18] Inequalities and lifestyle interpretation of population attributable fraction of modifiable risk factors for dementia [19]
Policy entry points	Primary prevention campaigns [27] Enrollment in clinical trials [13]	Action against inequalities (“primordial prevention”) [28] Primary prevention campaigns [27] Whole-population approaches [29]
Equity considerations	Likely to reinforce brain health inequities, unless specific actions are implemented [21, 22, 30]	Likely to reduce brain health inequities [21]
Regional origins	Global North, then transposed to other regions [13]	Global South [31, 32], where exposure to sources of ill brain health is higher
Implementation	Relatively simple to transpose to global contexts [33]	Reward structures in research and health policy favour fragmented short-termism [10]

The purpose of this comparison is not to encourage a sterile opposition between therapeutics and the scientific understanding of ADRD, but to build on a growing literature that highlights the pluralism that exists in expert approaches to conceptualising translational ADRD research [34, 35]. They are not mutually exclusive approaches, as they belong to the same multimodal prevention paradigm, and their compatibility is evidenced by extensive collaborations between proponents of the two approaches [33].

## Conclusion: re-defining and broadening what we mean by “translational research” in ADRD

The scope of translational research in ADRD is generally understood as “research that bridges the laboratory and clinical settings” [3]. Yet this narrow scope should be broadened to include broad health policy entry points. Rubio et al. [36] defined translational research as having three stages, T1, T2, and T3:

T1 research expedites the movement between basic research and patient-oriented research that leads to new or improved scientific understanding or standards of care. T2 research facilitates the movement between patient-oriented research and population-based research that leads to better patient outcomes, the implementation of best practices, and improved health status in communities. T3 research promotes interaction between laboratory-based research and population-based research to stimulate a robust scientific understanding of human health and disease. [p. 4, 36].

The wording here is important. In T1, the authors describe the translational process as “the movement between” basic and patient-oriented research (i.e. “bench to bedside”); in T2, this “movement” is between patient-oriented and population-based research leading to better health outcomes, but it is a similarly linear process. Conversely, T3 is an “interaction”, which when understood in the light of the complexity of the multimodal diversity approach [10], reminds us that we do not need to conceptualise translational research as a “linear pipeline” [37]. Thus, we can use a combination of the T1, T2, and T3 definitions and the multimodal diversity approach [10] to propose a normative re-definition of translational research in ADRD as follows:

Translational research in Alzheimer’s disease and Alzheimer’s disease-related dementias should promote a multi-directional interaction between, and multimodal integration of, laboratory-based and diverse population research to achieve a robust, global understanding of brain health and disease that can inspire equitable public health policy and advance precision medicine.

Equitable policy is stressed before precision medicine for the reason that the most promising trends in ADRD are seen at the population level [8, 29]. That is, age-adjusted incidence of dementia in high-income countries has decreased by up to 13% per decade in post-World War II birth cohorts [38, 39]. These incidence reductions do not correspond to improvements in precision medicine approaches to brain health during that same period [40], suggesting that reductions in health inequalities in those same periods [41] such as improved access to education and healthcare are most likely to be responsible for reductions in ADRD in those populations in high-income countries [21]. There is good reason to believe that structural interventions based on health policy to reduce health inequalities [42] should reduce incidence of ADRD while the evidence base is built [21]. Moving forwards, translational research that can inspire equitable policy is an important pillar of an adequate public health response to ADRD and neurological disorders more broadly [43].

There are three major priorities for implementation based on this perspective:

1. *Broaden research and policy* Fund research into the social determinants of dementia [28]. Advocate for policy addressing structural and social determinants of ill brain health alongside lifestyle interventions, allowing for broad policy entry points [21].
2. *Integrate and align research* stress the integration of existing data within research initiatives [10] so that ADRD research meets the brain health needs of diverse populations [21]. This includes integrating data from different brain-body-environment interactions [10, 44] and the co-creation of research design [10, 33] .
3. *Develop diverse biomarkers* Validate so-called “brain age clocks” in diverse populations [17] and seek their approval for use in clinical research across different health systems [45].

## Data Availability

No datasets were generated or analysed during the current study.
